# Facilitators of and barriers to blood donation among voluntary non‐remunerated blood donors in sub‐Saharan Africa: A scoping review

**DOI:** 10.1111/vox.70013

**Published:** 2025-03-19

**Authors:** Austrida Gondwe, Effie Chipeta, Mina C. Hosseinipour, Bridon Mbaya, Adamson S. Muula, Victor Mwapasa, Patani Mhango, Princess Kaira, Tiyamike Nthani, Sydney Puerto‐Meredith, Natasha Nsamala, Emmanuel Singogo, For the Malawi BLOODSAFE Program

**Affiliations:** ^1^ UNC Project‐Malawi, Tidziwe Centre, Kamuzu Central Hospital Lilongwe Malawi; ^2^ Kamuzu University of Health Sciences, Centre for Reproductive Health Blantyre Malawi; ^3^ Division of Infectious Diseases University of North Carolina at Chapel Hill School of Medicine Chapel Hill North Carolina USA; ^4^ Malawi Blood Transfusion Service Blantyre Malawi; ^5^ Kamuzu University of Health Sciences, School of Global and Public Health Blantyre Malawi

**Keywords:** barriers, blood donors, facilitators, sub‐Saharan Africa

## Abstract

**Background and Objectives:**

In many countries, including Africa, the widespread gap between national blood requirements and actual blood supplies contributes to unnecessary deaths. This scoping review explores common facilitators and barriers to blood donation in sub‐Saharan Africa (SSA) and strategies that are used to increase and maintain repeat blood donation.

**Materials and Methods:**

We conducted a scoping review of facilitators and barriers to blood donation in SSA. We searched PubMed, Ovid MEDLINE, Ovid EMBASE and COCHRANE to identify relevant papers. Of the 2225 articles screened by title, abstract and papers published after 2010, 37 were included in the final full‐text screening. Article quality for inclusion was assessed on the basis of a predefined eligibility and inclusion checklist. We analysed all papers that reported barriers and facilitators to blood donation in Africa.

**Results:**

The review included 11 studies. The common facilitators for blood donation reported were altruism, the opportunity for disease testing, friendly recruitment strategies and future easy access to blood at hospitals when needed. We identified the following barriers: lack of knowledge, awareness of blood donation and place of donation, fear, ineffective incentives, bad service experiences and religious and cultural beliefs.

**Conclusion:**

Findings of this review can be used to propose better strategies for improving blood donation in SSA. Strategies that encourage blood donation can be leveraged and implemented, while those that discourage blood donation can be adapted to better achieve an adequate supply.

**Highlights**

- In sub‐Saharan Africa, friendly blood donor recruitment strategies should be encouraged to leverage people's altruism and the health benefits associated with donating blood.
- Myths about blood donation, fuelled by cultural and religious beliefs, remain a crucial barrier to recruiting more voluntary non‐remunerated blood donors.
- Blood donation campaigns aimed at educating and raising awareness about blood donation should continue.

## INTRODUCTION

Globally, blood collection agencies increasingly face challenges in recruiting and retaining adequate numbers of blood donors [1]. Africa, in particular, faces medical conditions that often lead to anaemia, resulting in high blood transfusion needs [1, 2]. Moreover, the quantity of safe blood supply is insufficient to meet the high demand for blood transfusions in medical emergencies and treatment centres across Africa [3]. According to the World Health Organization (WHO), voluntary non‐remunerated blood donation (VNRBD) is the most reliable approach to ensuring a sufficient supply of safe blood to meet national requirements for blood transfusions [4]. Although blood donations from VNRBD increased by 10.7 million from 2008 to 2013 [4], sole reliance on VNRBD does not adequately address the blood supply shortage in many African countries [5]. In sub‐Saharan Africa (SSA), research suggests that altruism is the most common motivator for blood donation, while fear, lack of knowledge and discouraging cultural and religious beliefs are major deterrents [6].

Literature indicates that substantial progress has been made to achieve 100% VNRBD despite the high prevalence of human immunodeficiency virus (HIV) in some of the SSA countries (Namibia, Zambia, Mauritius and Botswana) [7]. In South Africa, more than a quarter of a million individuals are regular blood donors, yet only 10% of them are Black, despite Black people comprising 80% of those in need of transfusions [8]. In 2020, Tanzania faced a shortage of more than 300,000 blood units, and 15% of the collected units were not approved for various reasons, further reducing the available supply of usable blood [9]. Additionally, Malawi is experiencing a serious problem with inadequate supply of VNRBD and a shortage of safe blood supplies due to the high burden of (HIV), hepatitis B virus (HBV), hepatitis C virus (HCV), malaria and other infections in the general population [6]. For example, a study in Malawi hospitals revealed that hospital blood banks often ran dry, and communities struggled to access safe and adequate blood [10]. The Malawi Blood Transfusion Services (MBTS) has a low capacity to retain VNRBDs. However, VNRBDs from low‐risk population groups are crucial, contributing to 67% of the blood supply [10]. To promote blood donation, MBTS has adopted various strategies, such as hosting community engagement events, maintaining regular donors through incentives and establishing school programmes to encourage secondary school students to donate blood [10, 11].

Reviews focusing on the facilitators and barriers to blood donation in Africa are limited. Previous reviews conducted on motivators and deterrents of blood donation in Africa included all types of study designs regardless of the publication year [12, 13]. Understanding how SSA has progressed in increasing VNRBDs requires identifying effective strategies based on recent evidence about facilitators and barriers. Various reviews on this topic have been conducted in Western countries and included African populations, such as migrants residing in those countries [11, 14, 15, 16]. Thus, conducting a scoping review that includes studies and populations from Africa will help synthesize evidence to understand barriers and facilitators to maintaining repeat blood donors in Africa. Moreover, understanding the motivations and strategies of VNRBDs is critical to effectively engaging these donors.

## MATERIALS AND METHODS

### Search strategy

This scoping review was conducted following the Preferred Reporting Items for Scoping Reviews and Meta‐Analyses extension for Scoping Reviews (PRISMA‐ScR) guidelines (Table S2) and was registered in PROSPERO (registration #: CRD4202125439, https://www.crd.york.ac.uk/prospero/display_record.php?ID=CRD42021254390).

We developed a detailed systematic search strategy in collaboration with a librarian and information specialist. The search strategy was tailored to review articles indexed in medical and nursing databases, including PubMed, Ovid MEDLINE, Ovid EMBASE and COCHRANE.

A combination of the following search terms was used in PubMed to retrieve relevant articles on facilitators and barriers of blood donation: ‘Blood donation AND facilitators in Africa’, ‘Blood donation AND barriers in Africa’, ‘Facilitators OR motivators to blood donation in Africa’, ‘Barriers OR deterrents to blood donation in Africa’, ‘Blood donation in sub‐Saharan Africa’, ‘Repeat donation’, ‘Blood donation AND Malawi’, ‘Blood donation AND Africa’ and individual countries in SSA. These search terms were created following Sample, Phenomenon of Interest, Design, Evaluation, Research type for Scoping reviews (SPIDER‐ScR). The SPIDER tool offers a systematic strategy for searching qualitative and mixed‐methods research studies [17]. Through PubMed, we screened papers that included only abstracts. For full‐text articles unavailable via PubMed, we used other databases, including Ovid MEDLINE, Ovid EMBASE and COCHRANE. Additionally, we utilized Google Scholar to locate papers that were not accessible through the aforementioned databases. The data bases were searched between January and March 2021, with the recent search executed in February 2024.

### Study selection

The review targeted studies conducted in SSA. Eligibility criteria were based on the SPIDER‐ScR approach, which considers the sample, phenomenon of interest, design, evaluation and research type.

The review focused on studies that investigated or reviewed the barriers and facilitators of blood donation. Inclusion criteria included original full research papers published in English between 2010 and 2024. The studies had to examine VNRBD within SSA. The population included males and females aged between 16 and 65 years. Eligible studies were qualitative or mixed‐methods in nature, as these methodologies provide an opportunity to understand participants' perceptions at a single point in time. Qualitative research for this purpose was defined by the Cochrane methods groups [18], which synthesizes qualitative evidence to explore how people experience or value different health conditions, treatments and outcomes.

We excluded (i) reviews and systematic reviews, (ii) studies that were purely quantitative, (iii) papers unavailable through open access, (iv) abstracts preceding papers, (v) theses and books, (vi) editorials, (vii) author responses and (viii) case reports and case series. We excluded papers that did not investigate facilitators and barriers to blood donation in SSA. Additionally, we excluded papers published before the year 2010, as they were not considered recent; also, papers in languages other than English were also excluded because of language limitations.

### Data extraction and quality assessment

Two authors (A.G. and E.S.) independently extracted a relevant list of full papers that met the selection criteria. These authors collaborated at all critical stages to eliminate selection bias. Co‐authors T.N. and B.M. independently reviewed the papers included in the final selection.

The following data elements were extracted from the selected studies: author details, year of publication, country where the study was conducted, study objectives, design, participant characteristics and research findings (blood donation, behaviour, intention, attitudes towards blood donation). Additionally, we examined whether the authors discussed limitations and addressed sources of bias. The synthesis of the papers was compiled in an Excel spreadsheet (Table SS1).

## RESULTS

### Eligible studies

**FIGURE 1:**
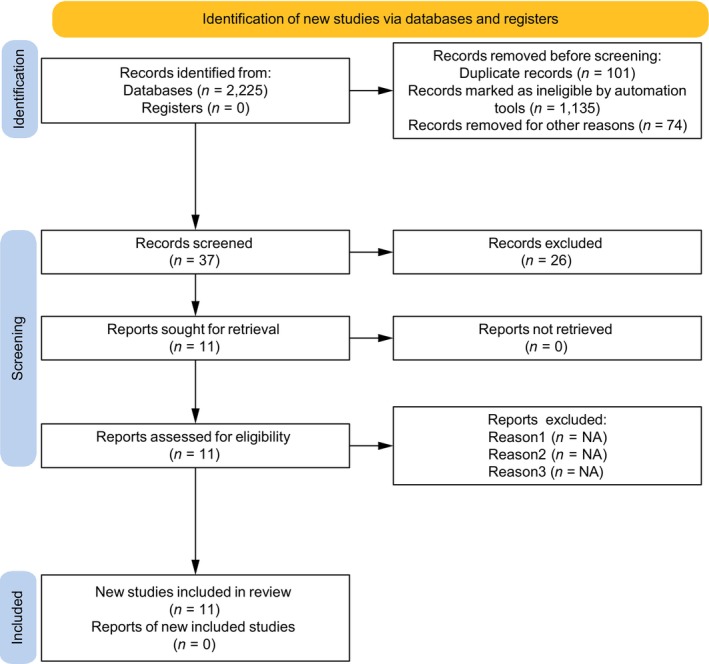
Preferred Reporting Items for Systematic reviews and Meta‐Analyses (PRISMA) flow diagram with details of the selection process of eligible studies. Excluded studies based on study design according to this paper are reviews, not open‐access papers, abstracts preceding papers, theses and books, editorials, author response, case reports and case series.

We retrieved a total of 2225 studies in our initial search. After screening based on the inclusion and exclusion criteria, 37 articles remained. Following the second screening, only 11 studies were included in the final review analysis (Figure 1). All of the included studies had been published between 2010 and 2024.

### Characteristics of included studies

The characteristics of the included studies are presented in Table 1. The sampling technique was detailed in all included studies. Out of the 11 studies, 3 used mixed methods [9, 19, 20] while 8 were qualitative [20, 21, 22, 23, 24, 25, 26, 27]. Two studies [22, 25] were conducted in hospital settings, whereas the rest were community‐based. Only two studies [21, 25] addressed issues of bias, and one study focused solely on barriers to blood donation, excluding facilitators of blood donation [28]. Additionally, two studies [24, 25] discussed study limitations, and one study [26] discussed the principal investigator's role in the methodology.

**TABLE 1:** Included and excluded papers.

Author	Country	Objective/aim	Study design	Participants	Barriers	Facilitators
Koster et al. (2011)	Cameroon	To better understand local attitudes towards blood donation and transfusion, to identify factors that motivate and deter blood donation	Direct observation, IDIs, FGDs (total not mentioned) and a simulation exercise	Clinical and laboratory staff, patient relatives, blood donors, secondary school students and community members (not mentioned in the paper	Fear of blood screening for HIV during blood donation. Fear of donated blood being used to cause harm to the donor through witchcraft. Fear that the donated blood will not be given to a family member	Charity or altruism (because blood itself was seen as a gift from God). Compensation in terms of cash, transport and food if someone travelled to donate blood
Muthivhi et al. (2015)	South Africa	To evaluate motivators and deterrents to blood donation among Black South Africans	13 FGDs	A total of 97 Black South Africans, stratified by age and geographic location	Low self‐efficacy, life style barriers, not enough blood, low involvement, inconvenience, lack of marketing communications, ineffective incentives (inadequate), lack of knowledge, negative service experience, fear, ineligible health conditions, negative attitudes, personal values	Convenience of collection site, pro‐social motivation, personal values, perceived need for donation, incentives and social norms
Nyambiya et al. (2020)	Zimbabwe	To identify and describe the behavioural beliefs underlying adults' blood donation intentions in Harare	Semi‐structured questionnaire	32 men and women residing in Harare, Zimbabwe	Traditional beliefs (donated blood can transmit evil spirits and bad behaviours, Satanism, can be used for rituals and that blood is not supposed to be shared), religious beliefs, charged for donated blood when one is sick, no adequate screening, adverse reactions, can negatively affect the one donating and the recipient and difficulties in regaining blood	Saves life, religious and social responsibility, chance to get tested for diseases, adopting more health behaviours, more blood is recovered, donating blood is safe, future use and accessibility
Rolseth et al. (2014)	Cameroon	To assess the characteristics of previous and potential blood donors by exploring the religious beliefs, and knowledge and understanding of blood donations among individuals present at a district hospital	In‐depth semi‐structured interviews (14 interviews)	41 IDIs with over 18 years, community members at Adamaoua region	Lack of awareness about blood donation, insufficient amount of blood, fear (of fatigue, dizziness, illness, not recovering after blood donation, of transmitting diseases to the recipient), lack of spouse's permission, busy schedules at work and ill health	Altruism, incentives (food and money), save a sick family member, save a friend, save life of someone and a positive attitude to supply to blood bank
Boahen et al. (2013)	Ghana	To determine what cultural beliefs and traditional practices might affect attitudes to blood draw	12 FGDs and 8 in‐depth interviews	12 FGDs and 8 in‐depth interviews among community members in Kintapo district of Ghana	Unpleasant experiences following blood draw, blood could be used for rituals and could lead to serious health consequences and fear of blood donation	Not mentioned
Baidoo et al. (2024)	Ghana	To explore the perspectives and experiences of young adults regarding blood donation processes	Mixed methods (number not specified for quantitative and qualitative)	382 participants	Lack of education on blood donation, fear of post‐donation health, issues associated with lack of privacy at blood collection centres	Intrinsic benefits and issues associated with disease risks
Ashapala (2022)	Namibia	The aim was to explore and describe the factors contributing to the low number of blood donors among the employed residents of the Oshatumba village, Oshana Region, Namibia	Qualitative (15 in‐depth semi‐structured interviews)	15 participants	Religious beliefs, lack of information, misconceptions associated with blood donation, long distances to blood donation centres	Not reported
Finda et al. (2022)	Tanzania	To explore awareness and perceptions of voluntary blood donation	Mixed methods (253 questionnaires and 4 FGDs)	253 for quantitative and 4 FGDs with 24 participants	Lack of knowledge of donation centres, fear of losing too much blood, fear of being anaemic, fear of knowing health status	To help family members, help friends, just for the sake of donating
Murtagh et al. (2021)	Uganda	To understand the factors that impact a person's decision to give blood and to inform public health campaigns that seek to promote donation	Qualitative (50 IDI and 22 KIIs)	50 IDIs 22 KIIs	Fear of needles, fear of losing blood, lack of awareness, lack of access to blood donation drives	Altruism, civic duty, opportunities for disease testing
Checkley et al. (2019)	Uganda	To gain insight into the community and hospital factors that contribute to the observed insufficient supply of blood units available for transfusion at a regional referral hospital in rural Eastern Uganda	Mixed methods using questionnaire but not specified for qualitative method	82 interviews altogether No specification for qualitative interviews	Lack of information, sickness and lack of food security	Altruism and to obtain blood in future
Karugaba et al. (2021)	Uganda	To explore barriers of blood donation among students of Mbarara University of Science and Technology	Descriptive qualitative study (4 FGDs)	24 participants	Side effects, inadequate knowledge, fear of needles, busy schedules, existing health conditions, culture, religion and inconvenient site	Not reported

*Note*: FGD is a facilitated discussion held with a small group of people who have specialized knowledge or interest in a particular topic.

Abbreviation: FGD, focus group discussions; HIV, human immunodeficiency virus; IDIs, in‐depth interviews; KII, key informant interview.

### Facilitators for blood donation

A summary of the motivating factors for blood donation is presented in Table 2. The most commonly reported facilitators included religious and social responsibility, altruism, incentives, the opportunity for disease testing, collectivism and future use. Altruism was reported in almost all studies as the main driver for blood donation [11, 19, 21, 23, 29]. Many participants felt that donating blood serves as a social responsibility for the communities in which they live. For others, donating blood is about the desire to have a positive impact in the community, described as ‘pro‐social motivation’. For others, there is ‘collectivism’, which is the motivation to help others, including family members, friends and the community [9, 21, 22, 23, 25].

**TABLE 2:** Facilitators to blood donation.

Motivator	Example/definition/intervention	Total studies (*n* = 7)
Pro‐social motivation (convenience and personal values) [23, 24, 25, 26]	The desire to donate blood to save lives in the community described as the main driver for donating blood. ‘It's a voluntary process that is meant to save lives; when the healthy person donates blood to those who need it for survival’ (P2, female, 25 years old) [3]	4
Altruism [19, 21, 23, 29]	Individuals' motivation to donate blood ‘The main reason why I've donated blood is to save people's lives’ (female first‐time donor) [21] Blood is a gift from God and it is good support others with blood	3
Collectivism (social responsibility) [3, 9, 21, 22, 23, 25]	Motivation to help others including family members, friends and the community. ‘Well, I think the benefits would be to help those who are in need, for example those involved in an accident … sick and desperately needs that blood’ (P30, male, 35 years) [21]	6
Obtain blood in the future [19, 24]	To obtain blood in future for oneself or family members when they are sick and need blood. ‘I the donor may also loose blood in future. If I lose blood and then get it through transfusion, if it then comes back to me, I will feel relieved … that I have been given back the life that I had lost’ (P04, female, 39 years) [24]	2
Incentives [23, 25]	Money and other recognition mechanisms for blood donors, i.e., T‐shirts money and any other gifts. ‘Incentives were the second most endorsed motivator, as evidenced by 20.1% of comments counted’ [21]	4
Health check [20, 21, 24, 26]	Blood donors get an opportunity for disease testing and are able to know their blood type. ‘It helps people to know their respective blood groups’ (P11, female, 30 years) [24] It also gives them opportunity to learn the food types to eat for health benefits (that can increase blood levels)	4

Religious beliefs were another significant motivator. For example, in Uganda, the majority of the participants (80%) reported that donating blood was enabling them to fulfil their religious belief … ‘My religion‐based motivation encourages me to consider what constitutes true religion. True religion has love, kindness, helpfulness’ [21]. In Zimbabwe, religious and social responsibility were the primary driver for blood donation [24]. Some participants reported that either they or their family members might need blood in the future [19, 24].

Awareness‐raising and recruitment strategies were mentioned as ways of encouraging people to donate blood [3, 20]. Among the promotion strategies, general advertising and direct marketing were mentioned more frequently than conducting sensitization meetings [3, 20, 23]. Some blood donors in Guinea, Cameroon and South Africa were motivated by the incentives and the recognition they were receiving through awards [23, 25, 26]. The common incentives reported were money, T‐shirts, food (lunch, drinks), transport reimbursement and other gift items [23, 25]. Additionally, opportunities for testing diseases (syphilis, HIV and malnutrition) as they present for blood donation were an important aspect for many blood donors, as well as the opportunity to know their blood group type [20, 21, 24, 26].

Other factors included adopting healthier behaviours through health education provided during blood donation activities [23]. For example, participants cited education on the right food to eat to maintain high haemoglobin levels in their bodies … ‘You will be benefitting in the process because when you will be eating fruits and drinking lots of fluids, you will be restoring your blood so that it becomes healthier, more than what it was … Sometimes it's a blessing in disguise’. Participants willingly donating blood felt that they should be incentivized after each donation so as to further motivate them [9, 23, 24, 25].

### Barriers to blood donation

Several barriers to blood donation were reported (Table 3). The most common included lack of knowledge and awareness [3, 19, 24, 29, 30, 31, 32], fear [3, 9, 20, 22, 27, 28], ineffective incentives [29], negative service experiences [23, 27], spiritual/religious factors [3, 21, 24, 28] and cultural perceptions [3, 21, 24, 27, 28] as well as negative attitudes towards blood donation [3, 23, 24, 27].

**TABLE 3:** Barriers to blood donation.

Donation barrier	Example/definition/intervention	Total studies (*n* = 7)
Lack of knowledge and awareness about blood donation and long distances [3, 20, 21, 25, 29]	No outreach programmes about blood donation, absence of marketing communication ‘… most people are not sensitized on what the blood is going to be used for and what are the consequences for donating blood …’ (female, faculty of medicine, FGD 1) [23, 28]	5
Fear [3, 9, 20, 21, 22, 27]	That blood may be sold than given to patients. ‘I also know that some of them sell blood to the patients, … so why will I go and donate if they're going to sell it to the patient’ (FGD participant 6) [20] That the process of drawing blood may be painful due to the needles ‘… donating blood using a needle …, to me I have a phobia for needles …’ (male, faculty of science, FGD 2) [28] That one may have side effects and get sick after donating blood ‘… students were seriously collapsing after donating blood and I was there once when I developed this phobia and I felt like not giving blood anymore …’ (ale, faculty of medicine, FGD 1) [28] That donating blood can negatively affect the donor's health ‘After donation of blood you can become vulnerable to infections’ (P5, male, 18 years old) [20] Fear of knowing serological status (being tested for other infectious diseases such as syphilis, HIV and nutrition status)	7
Serious health consequences /unpleasant experiences after blood draw/adverse effects	One may feel some dizziness and become sick after the blood is drawn from the body. ‘I saw some of them feeling weak after donating blood’ (P05, female, 46 years) [27] Faced with major health challenges during previous blood donation	3
Anxiety [3, 21, 24]	A lot of hearsay about blood donation. ‘We have religions in this country where they do not allow you to receive blood from someone else’—female first‐time donor	3
Long distances to donation centers/inconvenience [3]	Timing of blood donation, busy schedules at work and unknown places to donate blood ‘The distance to the donation site is very long and tiresome, and we lack transport to donate blood’ (P9, female, 36 years old) [3] Married women not having consent from husbands to donate blood ‘The husband is the head of the family; I cannot go to donate blood without his consent if he is not home’ [27]	1
Ineffective incentives [23]	There are no incentives at all to those who donate blood ‘… why would I be going there instead of hustling money somewhere?’ [23]	1
Cultural perceptions [3, 21, 24, 27]	Cultural perceptions about donating blood that blood will be used for rituals. ‘I want to donate but it is taboo in my culture so I am not really up for it’ (P3, female, 55 years old) [44]	4
Negative past experiences [21, 23, 27]	Long waiting time to donate blood, lack of privacy when your blood has some diseases and when someone has insufficient blood due to illness ‘When I was in secondary school, something happened. A student in the process of donating blood collapsed and they had to return her blood and even add her more blood. So, I'm really scared’—Female non‐donor	3
Negative attitudes [3, 23, 24, 27]	Health workers drawing blood should be nice to potential blood donors ‘Workers need to create good relationship with donors to make the environment conducive for the people to come and to continue donating blood’ (P11, female, 31 years old) [3]	3

Abbreviation: FGD, facilitated group discussion.

Studies that mentioned lack of knowledge and awareness (no outreach programmes) regarding importance of blood donation and venue (where to donate blood) suggested that improving education about blood donation would likely increase participation [21, 25, 29]. For instance, participants reported lack of blood donation campaigns, including posters and information as well as education and communication (IEC) materials on blood donation [3, 19]. Many community members were unaware of where and how to donate blood: ‘Some of us have not realized the role of giving out blood, so maybe I'm not informed, and this makes me run away from donating …’ [24].

Studies that mentioned fear further defined how fear impacted blood donation at different levels [3, 9, 20, 21, 22, 27, 28]. For instance, for some it was fear of serious health consequences after blood draw and unpleasant experiences after blood draw. Anxiety and adverse effects also demotivated people from donating blood. Some were afraid that their blood might be sold or given to people other than their family members. Additionally, fears of painful needles and superstitions about the use of donated blood further contributed to unwillingness to donate blood.

Religious and cultural perceptions further discouraged blood donation in countries such as Cameroon, Uganda, Namibia, Zimbabwe and Ghana [3, 22, 24, 27, 28]. Some religious groups in Zimbabwe, Guinea and Uganda believed that donating blood was a sin or that blood should not be shared [3, 21, 24]. Almost all papers reviewed reported issues of mistrust, unsafe blood donation procedures, inadequate community engagement, inconvenience, negative service experiences, accessibility challenges, negative attitudes that donated blood can negatively affect the donor's health or donated blood can negatively affect the recipient's health, unavailability of screening tests and inadequate storage facilities related to agents collecting blood. In some papers, lack of incentives and not being approached contributed to failure to donate blood [26, 29]. One paper reported the long distance to the donation site as the major challenge to donating blood and suggested that people who travel from long distances should be given transport [22].

## DISCUSSION

This scoping review identified and analysed 11 individual studies that investigated facilitators and barriers to blood donation in SSA. This review has found that some of the motivating factors for donating blood are almost similar in many countries. Altruism (saving lives, giving back to the community, donating in response to the need for blood in future) and pro‐social behaviour were some of the most commonly reported facilitators for blood donation in SSA. Furthermore, participants were motivated to donate blood because of the benefits of healthcare services such as comprehensive blood testing, escaping long queues and religious and social responsibility. Common barriers reported include various forms of fear, lack of knowledge of how, why and where to donate blood and religious and socio‐cultural beliefs.

Our findings are comparable to those of previous studies that have reported altruism as the common motivator for blood donation [16, 33, 34, 35]. For example, a study in the United States analysed donor experiences, facilitators and barriers using YouTube content and found that content creators were motivated to donate blood again because it made them feel good. One donor expressed: ‘I definitely want to donate more blood because I think it's really awesome that one pint of blood is saving three people's lives. So I saved [through] people's lives and I'm really excited!’ [33, 34]. However, some studies found that altruism did not have a strong impact on repeated donation [34, 36]. A study called ‘The importance of need‐altruism and kin‐altruism to blood donor behaviour for Black and White people’ reported that kin altruism was not associated with blood donor behaviour while need altruism did predict blood donor behaviour and was lower in Black British people [37]. Additionally, the study found that Black people are more willing to donate to a stranger, but the barrier is a belief that their blood will not be used, consistent with the observation from a South African study [8, 37]. Across literature, the desire to save lives—whether family member, community or strangers—was the main drive behind the passion to donate blood [9, 16, 38, 39].

The findings of our review are in agreement with those that have reported the benefits of healthcare services—such as escaping long queues and an opportunity for a thorough medical checkup including knowing some one's blood type—as beneficial to frequent blood donors especially in African countries [13, 38, 40, 41]. This allows donors to receive healthcare services promptly and return to their daily activities sooner. One healthcare worker stated: ‘This strategy motivates more people to come and donate blood as frequently as possible and this leads to enough blood supply to our blood bank’ [21, 22, 23, 24]. Policy makers can capitalize on this strategy, as it is not easy to receive timely health care in Africa due to congestion and shortage of service providers.

Although not frequently reported, the mention of incentives, whether monetary or non‐monetary, highlights the importance of recognition, even among those willing to voluntarily donate blood. For example, a review by Klinkenberg et al. of sub‐Saharan African migrants and minorities in Western high‐income countries revealed that special recognition or awards (donors 11.0%, non‐donors 13.7%) and receiving free gifts (donors 6.3%, non‐donors 9.1%) may motivate blood donation [11]. In some countries, incentives were mentioned among the non‐regular blood donors, but their impact was minimal [11, 23]. For example, a study examining the use of medical scheme benefits as an incentive for individuals who donated twice within a year reported that 83% of participants had donated more than twice annually, suggesting that self‐worth outweighed the offered benefit [42]. Additionally, there were disparities among the reviewed studies concerning blood donors' willingness to be compensated. Similarly, a study on the determinants of blood donation willingness in European countries by Huis et al. showed that different countries had different perceptions regarding what motivates people to donate blood [15]. This finding points to an in‐depth analysis and investigation of how incentives may influence blood donation and maintain repeat blood donors among some populations in Africa.

The findings of this review are inconsistent with those in the literature showing that blood donation in Africa is generally affected by prejudices, misconceptions and cultural differences [9, 11, 14, 15, 16, 19, 23]. Africa is a continent with diverse cultures that may influence blood donation decisions in some countries. Understanding specific cultural beliefs can help identify targeted strategies that may increase blood donation. In Uganda, the study found that a person from a royal family does not shed blood, hence cannot donate blood as one person revealed … ‘in my culture actually we all know that once you belong to the royal family you are not supposed to shade blood, loose blood anyhow like that, so we do not donate’ [28]. This is in contrast with a Nigerian study among the voluntary blood donors, which revealed that cultural factors do not affect voluntary blood donation in the area, as majority of the respondents believed that cultural practices do not forbid blood donation or expose the donor to witchcraft [39].

Although being religious is one of the motivators for blood donation, understanding certain religious beliefs is crucial, as they also affect the uptake of general health services in Africa including blood transfusion [3]. Our findings that religiosity and a sense of social responsibility motivated donors to keep donating blood are consistent with those of other studies that have reported similar results [7, 16, 34, 36]. In contexts where religious beliefs influence blood donation practices, it is important to identify effective strategies to encourage members of religious communities to donate blood. In Pakistan, religious believers (71%) believed that donating blood was a religious duty [43].

A lack of knowledge, such as not understanding the importance of donating blood and where to donate blood, has negatively impacted donor recruitment, leaving some potential donors unreached. These barriers were similarly reported in a Nigerian study by Waheed et al., who revealed that a lack of information and understanding about the need for and process of blood donation, among other factors, were the most frequent reasons discouraging individuals from donating blood [44]. This suggests the significance of providing information to both prospective and current donors to ensure the initiation and continuation of blood donation. Those responsible for recruiting donors in SSA should design interventions aiming at information dissemination that effectively penetrates to the right populations, thereby dispelling misconceptions about blood donation. Literature has suggested that raising awareness through different platforms, including campaigns, social media, news advertisements and radios, can facilitate blood donation [9, 11, 23]. Although awareness is highly recommended, Finda et al. argued that in western Tanzania nearly half of the respondents in two districts were aware of voluntary blood donation services, but only about a quarter reported ever donating blood [9]. Similar findings were observed in a study conducted in India, where 87.3% of community members surveyed had never donated blood despite having adequate knowledge of the services [45]. These observations highlight the need for a multifaceted approach when designing recruitment strategies to increase blood donation rates, by ensuring that other influential factors are not overlooked. However, WHO recommends community sensitization and mobilization as key strategies for increasing awareness about blood transfusion [46].

Global literature is inconsistent with our findings, showing that there are different types of fear associated with blood donation [8, 12, 16, 24, 45, 47, 48]. For instance, a meta‐analytic study by Bednal et al. found that fear was the most commonly identified barrier, and individuals who made subsequent donation attempts were less likely to report a fear of fainting or dizziness (17.9% vs. 26.2%), fear of needles or pain (13.5% vs. 21.1%) or concerns about being unsuitable donors due to personal health issues (0.4% vs. 3.1%) compared to those who did not attempt to donate [16]. Fears and misconceptions around blood donation are oftentimes linked to a lack of proper knowledge and information. Thus, providing adequate education and thorough sensitization to address these concerns may possibly motivate people to overcome their fears and start donating blood. Similar to our findings, other studies have reported health system‐related barriers such as long distances to the donation site and health workers' bad attitudes [8, 36].

Understanding people's perceptions and challenges related to blood donation in Africa is crucial for developing effective strategies to enhance blood availability in the region's blood banks. This review included qualitative studies to synthesize people's experiences, fears and motivators to donate blood. Our findings suggest that governments and policymakers should prioritize intensifying community engagement through influential figures, such as faith and traditional leaders, to identify effective ways of encouraging voluntary blood donation. Additionally, integrating blood donation into routine health services, developing locally tailored messages and utilizing visual aids may significantly enhance awareness about voluntary blood donation.

The weakness of this review is that the methodology of some of the included studies did not specify whether the target population was either blood donors (first, second and regular) or non‐blood donors. This lack of clarity makes it difficult to determine whether participants were reporting about their own experiences or those of others. However, a key strength of this review is its inclusion of eight qualitative studies that provide culturally relevant contexts. Additionally, all the reviewed studies were conducted in Africa, providing a comprehensive perspective on how blood donation is perceived, which can inform evidence‐based strategies for decision makers to improve blood donation practices.

In conclusion, this review has highlighted both the facilitators and barriers to blood donation in SSA. Facilitators such as altruism, incentives, collectivism and donating blood for future benefits need to be promoted to encourage blood donation in SSA. However, issues of lack of awareness, mistrust, fear and religious and spiritual factors are important for policymakers, including government and agencies, to address. Strategies that encourage blood donation can be adopted and implemented, while barriers to blood donation can be used to conduct further research to gain more insights and ultimately improve blood donation practices.

## CONFLICT OF INTEREST STATEMENT

The authors declare no conflicts of interest.

## Supporting information


**Table S1.** Synthesized studies.

**Table S2.** Preferred Reporting Items for Systematic reviews and Meta‐Analyses extension for Scoping Reviews (PRISMA‐ScR) Checklist.

## Data Availability

Data sharing is not applicable to this article as no new data were created or analysed in this study.
