# Supplementary material for: Facilitators of and barriers to blood donation among voluntary non‐remunerated blood donors in sub‐Saharan Africa: A scoping review
Source: Vox Sang. 2025 Mar 19;120(6):546–56. doi: 10.1111/vox.70013 (PMC12187535; doi:10.1111/vox.70013)
Supplement: Supplementary file 1 — Table S1. Synthesized studies. [file VOX-120-546-s002.docx]

**Table S1: Synthesized studies**

| **Author** | **Country** | **Objective/aim** | **Study design** | **Participants** | **Barriers** | **Facilitators** | **Limitations indicated** | **Bias explained** |
| --- | --- | --- | --- | --- | --- | --- | --- | --- |
| Koster J eta l (2011) | Cameroon | To better understand local attitudes towards blood donation and transfusion, to identify factors that motivate and deter blood donation | Direct observation, in-depth interviews, FGDs and a simulation exercise | Clinical and laboratory staff, patient relatives, blood donors, secondary school students and community members | Fear of not being able to tolerate the loss of blood and/or replace it. Fear of blood screening for HIV during blood donation. Fear of donated blood being used to cause harm to the donor through witchcraft. Fear that the donated blood will not be given to a family member | Charity or altruism (because blood itself was seen as a gift from God). Compensation in terms of cash, transport and food if someone travelled to donate blood | yes | no |
| Muthivhi TN eta l, 2015 | South Africa | To evaluate motivators and deterrents to blood donation among Black South Africans | 13FGDs | A total of 97 Black South Africans, stratified by age and geographic location | Low self-efficacy, life style barriers, not enough blood, Low involvement, Inconvenience, lack of marketing communications, Ineffective incentives (inadequate), lack of knowledge, negative service experience, fear, ineligible health conditions, negative attitudes, personal values. | Convience of collection site, Prosocial motivation, personal values, perceived need for donation, indirect reciprocity, promotional communication, Incentives and social norms | yes | no |
| Murtagh et al, 2020 | Uganda | To understand factors that impact a person’s decision to give blood and to inform public health campaigns that seek to promote donation. | 50 semi structured in-depth interviews and 22 key informant interviews | 50 Uganda Blood transfusion staff and 22 Uganda Red Cross Staff | Fear of needles, myths and misconceptions about donating blood, fear of learning disease status, lack of access, lack of knowledge, buying blood for their relatives when they are sick, negative past experience, religion and ineligibility (haemoglobin too low). | Altruism, blood credit, blood disease testing, positive past experiences, material incentives and health improvement | no | no |
| Nyambiya TE et al, 2020 | Zimbabwe | To identify and describe the behavioural beliefs underlying adults’ blood donation intentions in Harare. | Semi structured questionnaire | 32 men and women residing in Harare, Zimbabwe | Traditional beliefs (donated blood can transmit evil spirits and bad behaviours, Satanism, can be used for rituals and that blood is not supposed to be shared), Religious beliefs, charged for donated blood when one is sick, no adequate screening, adverse reactions, can negatively affect the one donating and the recipient and difficulties in regaining blood | Saves life, religious and social responsibility, chance to get tested for diseases, adopting more health behaviours, more blood is recovered, donating blood is safe, future use and accessibility | no | no |
| Rolseth S et al, 2014 | Cameroon | To assess the characteristics of previous and potential blood donors by exploring the religious beliefs, and knowledge and understanding of blood donations among individuals present at a district hospital. | In-depth semi structured interviews | 41 in-depth interviews with over 18 years, community members at Adamaoua region | lack of awareness about blood donation, insufficient amount of blood, fear (of fatigue, dizziness, illness, not recovering after blood donation, of transmitting diseases to the recipient), lack of spouse's permission, busy schedules at work and ill health | Altruism, incentives (food and money), save a sick family member, save a friend, save life of someone and just a positive attitude to supply to blood bank |  |  |
| Ronse M et al, 2018 | Guinea | To describe what motivated or deterred Ebola Virus Disease survivors to donate convalescent plasma | Interviews, observations and FGDs | Participants were those who were Ebola Survivors and those with social ties to ebola survivors. | Fear of the unknown for the first time donors, fear of knowing one's serological status, fear of the pain, needles, and physical weakening, especially when survivors felt they had not completely recovered from EVD or when they felt they were affected by poor nutrition, healthy risk | Incentives ( free testing for diseases such as syphilis, HIV, hepatitis B and C, transport reimbursement and phone credit) | no | no |
| Boahen O et al, 2013 | Ghana | To determine what cultural beliefs and traditional practices might affect attitudes to blood draw. | FGDs and in-depth interviews | 12 FGDs and 8 in-depth interviews among community members in Kintapo district of Ghana | Unpleasant experiences following blood draw, blood could be used for rituals and could lead to serious health consequences and fear of blood donation | Not mentioned | no | no |
| Karugaba et al, 2021 | Uganda | To explore barriers of blood donation among students of Mbarara University of Science and Technology. | Descriptive qualitative study (4 FGDs). | 24 participants. | Side effects, inadequate knowledge, fear of needles, busy schedules, existing health conditions, culture, religion and inconvenient site. | Not reported | yes | no |
| Ashapala D O, 2022 | Namibia | The aim was to explore and describe the factors contributing to the low number of blood donors among the employed residents of the Oshatumba village, Oshana Region, Namibia. | Qualitative (15 in-depth semi structured interviews) | 15 participants | Religious beliefs, lack of information, misconceptions associated with blood donation, long distances to blood donation centers. | Not reported | yes | no |
| Checkley et al, 2019 | Uganda | To gain insight into the community and hospital factors that contribute to the observed insufficient supply of blood units available for transfusion at a regional referral hospital in rural Eastern Uganda. | Mixed-methods using questionnaire but not specified for qualitative method | 82 interviews altogether and no specification for qualitative interviews | Lack of information, sickness and lack of food security. | Altruism and to obtain blood in future. | yes | no |
| Finda F et al, 2022 | Tanzania | To explore awareness and perceptions of voluntary blood donation | Mixed methods (253 questionnaires and 4 FGDs) | 253 for quantitative and 4 FGDs with 24 participants | Lack of knowledge of donation centers, fear of losing too much blood, fear of being anemic, fear of knowing health status | To help family members, help friends, just for the sake of donating | yes | no |
